# AntagomiR-19a Induced Better Responsiveness to Bortezomib in Myeloma Cell Lines

**DOI:** 10.22074/cellj.2021.7302

**Published:** 2021-10-30

**Authors:** Azam Kazemi, Saeid Abroun, Masoud Soleimani

**Affiliations:** A. Department of Haematology and Blood Banking, Faculty of Medical Sciences, Tarbiat Modares University, Tehran, Iran

**Keywords:** Antagomir-19a, Bortezomib, Multiple Myeloma, SOCS3

## Abstract

**Objective:**

Multiple myeloma (MM) is the clonal proliferation of neoplastic plasma cells in the bone marrow. Although bortezomib (BTZ) is a crucial drug for the treatment of MM, drug resistance is a major problem. OncomiR-19a plays an oncogenic role in many cancers, including MM; however, the function of miR-19a in the pathogenesis of MM and drug resistance has not been completely identified. The present research aims to investigate the inhibition of miR-19a by an antagomir to determine BTZ responsiveness, and determine if miR-19a can be a prognostic biomarker for MM.

**Materials and Methods:**

In this experimental study, viability and apoptosis of myeloma cells were analysed by the colorimetric 3-(4, 5-Dimethylthiazol-2-yl)-2, 5-Diphenyltetrazolium Bromide (MTT) and Annexin V/propidium iodide (PI) flow cytometry assays. Quantitative real-time polymerase chain reaction (qRT-PCR) was implemented to evaluate the expression levels of miR-19a, its targets *SOCS3, STAT3,* B-cell lymphoma 2 (*BCL-2*), *PTEN* and *CDKN1A* (anti- apoptotic and cell cycle related genes) at the mRNA level.

**Results:**

miR-19a was downregulated and exacerbated in transfected cells treated with BTZ. The rate of apoptosis in the myeloma cells after BTZ treatment considerably increased, which indicated an increase in the mRNA of *SOCS3*, *PTEN, BCL-2,* and *CDKN1*. A decrease in STAT3 was also observed.

**Conclusion:**

OncomiR-19a, as a biomarker, may induce better responsiveness to BTZ in myeloma cell lines through its targets *SOCS3, STAT3* and *PTEN*. In the future, this biomarker may provide new therapeutic targets for MM.

## Introduction

Multiple myeloma (MM) is a tumour of differentiated B cells from the germinal centre, plasma cells, within 10% of all haematologic neoplasms and is considered the second most commonly occurring non-Hodgkin lymphoma (1, 2). Over the last two decades, advancements in available treatments have increased the median survival time of patients with MM from three to six years. MM accounts for 2% of total cancer deaths and more than 20% of deaths due to malignancies (3). Although tremendous advancements have been made in new healthcare strategies in the past decade, to a great extent, this tumour is incurable and new therapies are required (4, 5). Despite the innovation and benefits of a new therapeutic strategy such as proteasome inhibitors, the clinical outcome of the patients aggravates and most patients with MM eventually relapse and engenders drug resistance (1).

The proteasome inhibitor, bortezomib (BTZ), is a crucial Food and Drug Adminisration (FDA)-approved drug for the treatment of MM, especially in patients diagnosed with relapsed and refractory MM (6). Although BTZ has a significant impact on MM treatment (5), drug resistance or relapse are two major challenges and patients with BTZ resistance have a poor prognosis (7, 8). Therefore, new therapeutic methods are urgently required to prevent BTZ resistance. In addition, a more profound molecular grasp of this cancer’s pathogenesis is required to recognize new molecular targets and present therapeutic agents suitable for patients (9). There is developing evidence that MM stems from the deregulation of noncoding RNAs (ncRNAs), which include microRNAs (miRNAs) (1). Recent studies show that MM is caused by interruptions in many different signalling pathways driven by miRNAs that are a class of ncRNAs about 18-22 nucleotides (nt) in length. These miRNAs act as master regulators of gene expression at the post-transcriptional level via RNA interference pathways (6). miRNAs are involved in many biological processes that include differentiation, senescence, survival and apoptosis (3, 6). Disturbances in miRNA regulation are accompanied by the pathogenesis of diseases such as cancer, and miRNA expression profiles have prognostic implications in numerous types of cancer (6). Altogether, miRNAs play a fundamental role as an oncogene and they operate as ‘oncomiRs’ if their targets are tumour suppressor genes (1). Therefore, controlling oncomiRs may be an effective treatment strategy.

The miR-17-92 clusters located in chromosome 13q31.3, including miR-19a, were the first oncomiRs discovered. Disturbances in the expression levels of miR-17-92 clusters result in malignant progression of MM (4, 5). miR-19a, a crucial component of the miR-17-92 cluster, is directly involved in myeloma pathogenesis and progression of MM (9). In addition, target genes of miR-19a are considered potential biomarkers of this disease (10). Compared with normal plasma cells, miR-19a is upregulated in patients with MM and in MM cell lines.

miR-19a can adjust the expressions of proteins essential for myeloma pathogenesis and include suppressors of cytokine signalling (*SOCSs*). miR-19a targets *SOCS3*, a potent regulator of the JAK-STAT pathway, which is followed by a considerable reduction in *SOCS3* mRNA together with enhanced activation of the *SOCS3* target, *STAT3* (9). Based on these findings, a strategy that can be developed to regulate aberrant expressions of miRNAs in cancer is the inhibition of upregulated miRNAs (1). Therefore, we assume that the use of mir-19a inhibitors (antagomir) could be a new treatment approach for MM.

B-cell lymphoma 2 (*BCL-2*) is one of the anti-apoptotic members of the *BCL-2* family that interacts with these proteins and in response to drug therapy, it determines cellular fate decisions and represents an attractive target for therapy (11, 12). *In vitro* studies indicated that *CDKN1A* might be an oncogene in lymphomas and plasma cell disorders, and these studies indicate that *CDKN1A* can act as a molecular target for drug developments (13). Although BTZ is used to treat MM, 60% of patients treated with BTZ experience resistance. Therefore, we intend to investigate the impact of antagomiR-19a on improving responsiveness to BTZ. The findings may show that miR-19a can be an effective biomarker for treatment response (9, 14).

## Materials and Methods

### Cell lines and cultures

In this experimental study, we purchased the RPM I8226 and U266 cell lines from Pasteur Institute of Iran (IPI), Tehran, Iran. The cells were grown in suspension in RPMI 1640 medium (Bio-Idea, Bio Idea Group, Iran) supplemented with 10% foetal bovine serum (FBS, Gibco-BRL, Germany), 100 mg/mL penicillin, 100 mg/mL streptomycin, and 2 mM L-glutamine (Bio-Idea, Bio Idea Group, Iran). The cells were maintained at 37˚C in an environment of 5% CO_2_ and 95% air, and were passaged twice per week. 

The present study was conducted with the approval of the Ethical Committee of the Tarbiat Modares University (IR.TMU.REC.1394.290).

### Reagents

BTZ (PS-341, Selleckchem.com, cat. no. S1013) was dissolved in 0.2603 mL DMSO to prepare a 50 mM stock solution and stored at -20˚C. The LentimiRa-off-has-miR-19a-3p vector (Applied Biological Materials, Inc., cat. no. mh30299) that included a green fluorescent protein (GFP) promoter, miRNA insert and kanamycin resistance gene was transformed in a DH5α E. coli strain, then isolated with a Qiagen plus Midi Plasmid Purification kit. The final product was stored at -20˚C until further use.

### *In vitro* cell culture and drug treatment 

The human myeloma cell lines RPMI 8226 and U266 were cultured in RPMI 1640 and the stock solution of BTZ was diluted in RPMI 1640 medium prior to use. RPMI 8226 and U266 cells were cultured in RPMI 1640 medium, then seeded at a density of 5×10^3^ cells in 96-well plates. These cells were treated with various working concentrations of BTZ, which was obtained from a 50 mM stock solution, in order to determine the half maximal inhibitory concentration (IC_50_) for each cell line.The concentrations of BTZ were based on approximate concentrations noted in the cell assay part of the BTZ datasheet, which were 0.5, 5, and 50 µM for U266 and 150, 450, 750, and 1050 nM for RPMI 8226. The BTZ concentrations and cells were mixed well in RPMI 1640 medium and 10% FBS, and incubated for 48 hours.

### 3-(4, 5-dimethylthiazol-2-yl)-2, 5-diphenyltetrazolium bromide colorimetric assay

We used a standard protocol to assess the inhibitory impact of BTZ on cell growth by the 3-(4, 5-dimethylthiazol-2-yl)-2, 5-diphenyltetrazolium bromide (MTT) assay. Briefly, cells from the 48-hour cultures were pulsed with 10 µL of 5 mg/ mL MTT in each well for at least four hours of the 48-hour cultures, followed by 100 µL of isopropanol that contained 0.04 N HCl. Absorbance was measured at 570 nm using a spectrophotometer and the results were expressed as the mean of three replicates, as a percentage of the control (100%). The extent of cytotoxicity was defined as the relative reduction in optical density, which correlated to the number of viable cells in relation to the control (100%). In order to decide the optimum dosage of the drugs for further studies, the cell viability was plotted in a graph and we calculated the IC_50_.

### Cell viability analysis

The effect of transfection on cellular viability was assessed by flow cytometry using propidium iodide (PI). PI can only pass through disordered areas of the membranes of nonviable cells and intercalate with DNA of the nuclei, emitting a red fluorescence light. PI solution was used with 1 mg/mL concetration by dissolving PI (Sigma, P 4170, Germany) in dH_2_ O. The PI solution was added in a final concentration of 2 µg/mL to 1×10^6^ cells in suspension, incubated in the dark for five minutes, then analysed by flow cytometry with an Attune NXT flow cytometer.

### Analysis of apoptosis

We assessed the level of apoptosis by annexin V/PI staining and flow cytometry with an Attune NXT flow cytometer in transfected cells that were treated with BTZ and in the untreated cells. The cells were washed in PBS and then in 1X binding buffer before they were resuspended in 1X binding buffer at 1×10^6^ cells/mL. Then, we added 5 µL of FITC-conjugated annexin V to 100 µL of the cell suspension and incubated this suspension for 10-15 minutes in the dark at room temperature. The incubated cells were washed with binding buffer and resuspended in it. Next, we added 5 µL PI staining solution (Sigma, P 4170, Germany) and analysed the cells with flow cytometry. 

### Prediction of *SOCS3* as a target of miR-19a

TargetScan (version 5) and PicTar were used to confirm that *SOCS3* is a target of miR-19a at its 3´UTR. TargetScan predicted the biological targets of miRNA by searching for the presence of conserved 7 and 8 base sites that match its seed region.

### Cell transfection

The cells were grown in RPMI 1640 medium without antibiotics prior to transfection. The U266 and RPMI 8226 cell lines were transfected by a final concentration of 2 µg of the pLenti-III-miR-Off-has-miR-19a-3p vector that contained GFP (Applied Biological Materials, Inc., Canada). Transfection of cells was performed using UltraCruz® Transfection Reagent (Santa Cruz Biotechnology, Inc., Germany). Briefly, before transfection, we prepared transfection reagent and the vector in Opti-MEM I reduced serum medium (Gibco, Germany) in accordance with the manufacturer’s protocol. The transfection reagent and plasmid were prepared in OPTI-MEM I medium and incubated at room temperature. Next, we added the plasmid reagent to the transfection reagent, vortexed it vigorously, and incubated the mixture for 20 minutes. A total of 6×10^3^ cells were added to the Eppendorf tube and were poured above mix dropwise to the cells, then the solution was incubated in the incubator for two hours. Each 30 minutes the tube was flicked by a fingertip. After the incubation, the cells were transferred to a six-well plate and incubated for 24-72 hours, followed by evaluation of GFP expression by an Attune NXT flow cytometer.

### Quantitative real-time polymerase chain reaction assessment of miR-19a expression

Total RNA was isolated from the untransfected, transfected, and BTZ-treated RPMI 8226 and U266 cells according to the TRIzol manufacturer’s protocol (InvitrogenTM, USA). A total of 2000 ng of RNA was reverse transcribed using specific miRNA stem-loop primers from Qiagen for miR-19a to generate cDNA using a Hyperscript Reverse Transcriptase First-strand Synthesis kit (GeneAll Biotechnology Co., Ltd., Korea). Snord47 was used as the internal control for normalization of miRNA expression. Quantitative real-time polymerase chain reaction (qRT-PCR) was performed with a SYBR® Premix Ex Taq™ miRNA RT-qPCR Detection Kit (Takara, USA, cat. no. RR820Q) using a Qiagen Rotor-Gene Q 5PLEX HRM Real-Time PCR. Table 1 shows the primer sequences used in this experiment. The PCR program cycling parameters were: 95˚C for 15 seconds, 58˚C for 30 seconds, and 72˚C for 30 seconds for 45 cycles. Data analysis was done by 2^-ΔΔCT^ to calculate the fold change for relative miR-19a expression compared to the untreated control.

### Quantitative real-time polymerase chain reaction analysis for the *SOCS3, STAT3, PTEN, BCL-2 *and *CDKN1A* genes

Total RNA was isolated from the untransfected, transfected, and BTZ-treated RPMI 8226 and U266 cells according to the TRIzol manufacturer’s protocol (InvitrogenTM, USA). A total of 2 μg of total RNA was reverse transcribed into cDNA using a Hyperscript Reverse Transcriptase First-strand Synthesis kit with oligo-dT primers in accordance with the manufacturer’s instructions (GeneAll Biotechnology Co., Ltd., Korea) for evaluation of the *SOCS3, STAT3, BCL-2, CDKN1A* and *PTEN* genes. *β-Actin* was used as the internal control. qRT-PCR was performed with the SYBR® Premix Ex Taq™ miRNA RT-qPCR Detection Kit (Takara, USA, cat. no. RR820Q) using a Qiagen Rotor-Gene Q 5PLEX HRM Real-Time PCR. The PCR program cycling parameters were: 95˚C for 15 seconds, 58˚C for 30 seconds, and 72˚C for 30 seconds for 45 cycles. Data analysis was performed by 2^-ΔΔCT^ to calculate the fold changes for the relative expressions of the above genes compared to the untreated control.

### Statistical analysis

Data was presented as mean ± standard deviation. The statistical analysis was performed with the Graphpad prism 8.4.0 software The mean values of two groups or multiple groups were compared by one-way analysis of variance (ANOVA). P<0.05 was considered statistically significant. Flow cytometric assays were analyzed with flowjo version 7.6.1. 

## Results

### Determination of the IC_50_ for bortezomib in the U266 and RPMI 8226 multiple myeloma cell lines 

**Fig.1:**
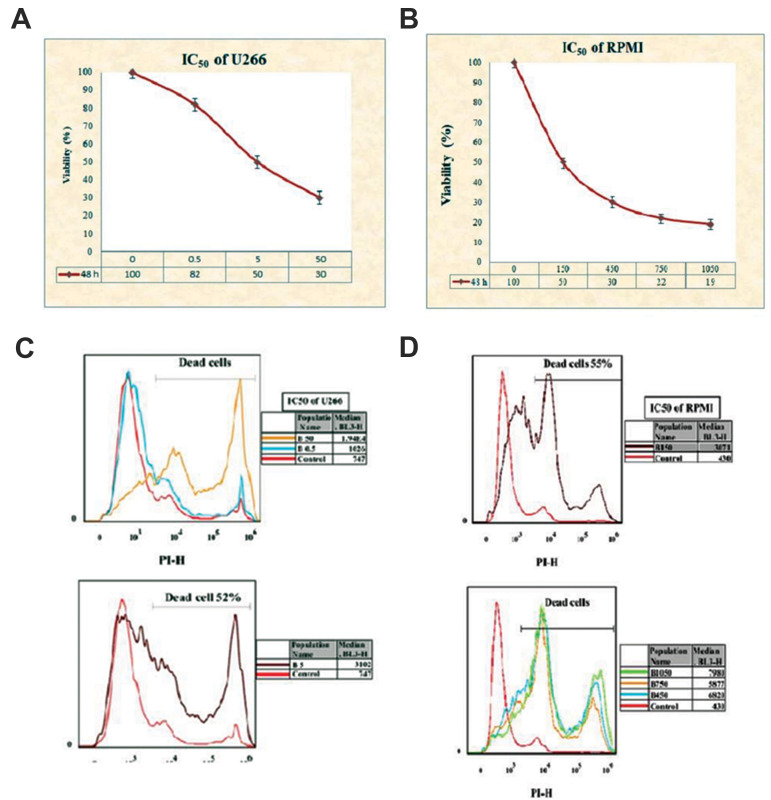
IC_50_ evaluation of the U266 and RPMI 8226 cell lines to optimize the concentration of bortezomib (BTZ). **A, B.** The U266 and RPMI 8226 cell lines were incubated with increasing concentrations of BTZ (0 to 50 µM) and (0 to 1050 nM), respectively, for 48 hours. Cell viability was assessed by the 3-(4, 5-Dimethylthiazol-2-yl)-2, 5-Diphenyltetrazolium Bromide (MTT) colorimetric assay. **C, D.** Treated U266 and RPMI 8226 cells were incubated with propidium iodide (PI) and the percentage of nonviable cells was assessed by flow cytometry.

We treated the RPMI 8226 and U266 cell lines with different concentrations of BTZ to determine the optimal IC_50_ to treat the cell lines with proper concentrations of BTZ throughout the analysis. The MTT assay and viability test with PI by flow cytometry showed that the optimized concentration of BTZ for the U266 cell line was 5 µM and it was 150 nM for the RPMI 8226 cell line (Fig .1A-D).

### Downregulation of miR-19a decreased cell viability and prevented proliferation of the myeloma cell lines

**Fig.2:**
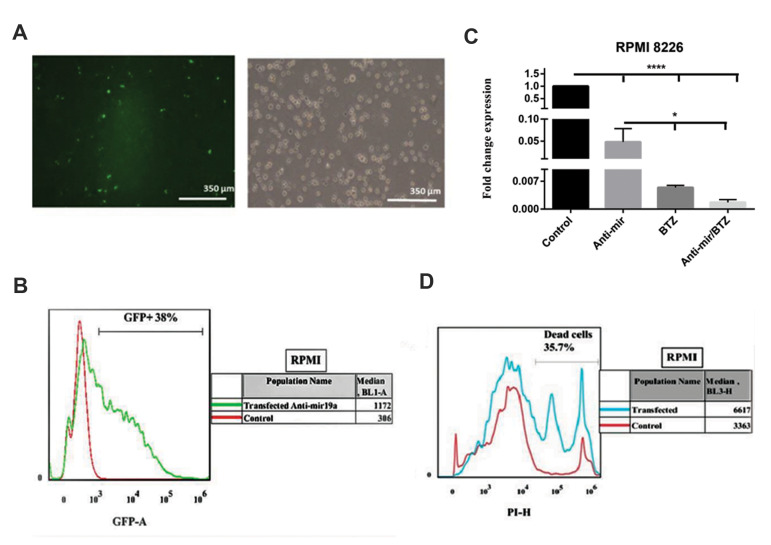
RPMI 8226 cells were transfected with the LentimiRa-off-has-mir-19a-3p vector. **A, B. **The transfection efficiency was assessed by fluorescence microscopy and flow cytometry through GFP fluorescence. **C. **miR-19a expression was assessed by quantitative real-time polymerase chain reaction (qRT-PCR) in the non-transfected RPMI cell line (control), transfected RPMI cell line with the LentimiRa-off-has-mir-19a-3p vector, treated RPMI cell line with bortezomib (BTZ) and treated RPMI cell lines with BTZ after transfection with the LentimiRa-off-has-mir-19a-3p vector The ratios of miR-19a were calculated relative to Snord47. Values are expressed as the mean ± standard deviation of three independent experiments. **D. **Evaluation of RPMI 8226 myeloma cell line viability at 72 hours after transfection. RPMI 8226 cells were transfected with the LentimiRa-off-has-mir-19a-3p vector, then incubated with propidium iodide (PI). Cell viability was assessed by flowcytometry. *; P<0.05 vs. the control and ****; P<0.001.

**Fig.3:**
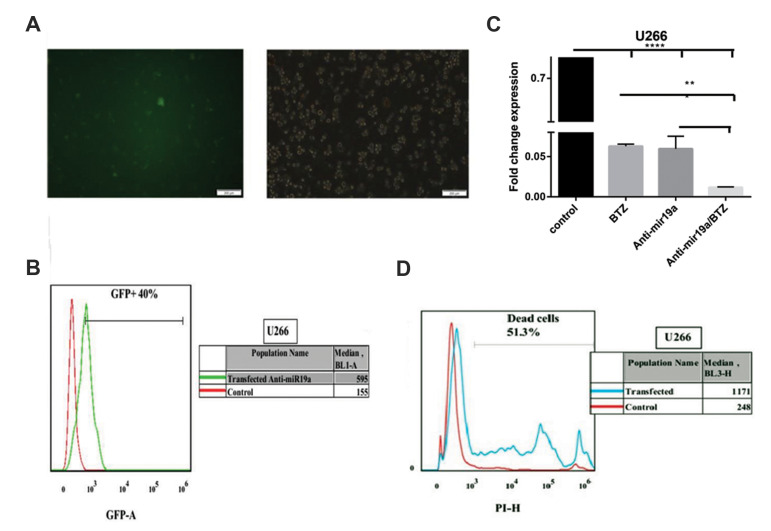
U266 cells were transfected with the LentimiRa-off-has-mir-19a-3p vector. **A, B. **The transfection efficiency was assessed by fluorescence microscopy and flow cytometry through GFP green fluorescence. **C.** Expression of miR-19a was assessed by quantitative real-time polymerase chain reaction (qRT-PCR) in the non-transfected U266 cell line (control), U266 cell line transfected with the LentimiRa-off-has-mir-19a-3p vector, U266 cell line treated with bortezomib (BTZ) and U266 cell line treated with BTZ after transfection with the LentimiRa-off-has-mir-19a-3p vector. The ratios of miR-19a were calculated relative to snord47. Values are expressed as the mean ± standard deviation of three independent experiments. **D.** Evaluation of U266 myeloma cell viability 72 hours after transfection. U266 cells were transfected with the LentimiRa-off-has-mir-19a-3p vector then the cells were incubated with propidium iodide (PI) and cell viability was assessed by flow cytometry after 72 hours. **; P<0.001 vs. the control and ****; P<0.0001.

We first evaluated the expression levels of miR-19a in the non-transfected (control) RPMI 8226 and U266 myeloma cell lines. After transfection of these cell lines with the LentimiRa-off-has-miR-19a-3p vector, the efficiency of transfection was monitored by GFP fluorescence as observed by fluorescent microscopy (Fig .2A, Fig 3A) and flow cytometry (Fig .2B, Fig 3B). The expression levels of miR-19a were determined in the transfected RPMI 8226 and U266 myeloma cell lines by qRT-PCR. As shown in Figures 2C and 3C, the expression level of miR-19a significantly decreased in the transfected group compared with the un-transfected myeloma cell line. The data showed that the antagomir-19a downregulated expression of miR-19a, a previously-known oncomiR. After transfection of the RPMI 8226 and U266 myeloma cell lines with the LentimiRa-off-has-miR-19a-3p vector, we evaluated cell viability after 72 hours with PI and the rate of viability was detected by flow cytometry (Fig .2D, Fig 3D). The data confirmed the possibility of the analyses of the cell lines within 72 hours after transfection, and indicated a decrease in cell viability because of miR-19a downregulation. When compared with the negative control group, this finding suggested that miR19a suppression decreased cell proliferation.

### Reduction in miR-19a expression in the myeloma cell lines after bortezomib treatment

We used qRT-PCR to evaluate the expression level of miR-19a after treatment with BTZ. Our data showed a reduction in its expression in the treated cells compared to the non-treated cells. miR-19a expression was also evaluated in cells transfected by the LentimiRa-off-has-miR-19a-3p vector. The data showed a substantial reduction in miR-19a expression after BTZ treatment compared to the non-treated and non-transfected controls (Fig .2C, Fig 3C). Cell viability evaluation after 72 hours with PI using flow cytometry showed that it was possible to evaluate every analysis on the transfected cell lines 72 hours after transfection. On the other hand, viability of the cell lines decreased due to downregulation of miR-19a (Fig .2D, Fig 3D).

### Anti-miR-19a increased susceptibility to bortezomib-induced apoptosis

**Fig.4:**
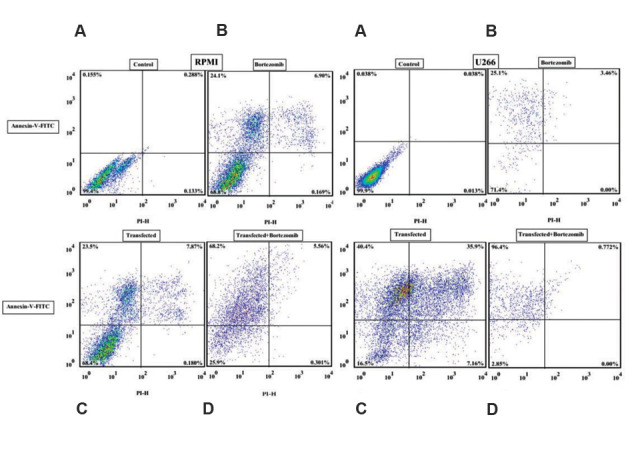
Evaluation of apoptosis by annexin V-FITC/propidium iodide (PI) staining and analysis by flow cytometry in RPMI 8226 and U266 myeloma cell lines. **A. **Untreated and nontransfected cell lines are the negative controls. **B.** After bortezomib (BTZ) treatment. **C. **After transfection with the LentimiRa-off-has-mir-19a-3p vector. **D.** After treatment of the transfected RPMI cell line with BTZ.

We investigated the effect of antagomir-19a on BTZ-induced apoptosis on the myeloma cell lines. The LentimiRa-off-has-miR-19a-3p transfected myeloma cells were incubated for 48 hours with BTZ (5 µM for U266 and 150 nM for RPMI 8226) followed by annexin V/PI staining and flow cytometry analysis to determine the percentage of apoptosis. The percentage of cells that underwent apoptosis increased after transfection in the RPMI 8226 (23.5% vs. 68.2%, P=0.0038) and U266 (25.2% vs. 96.4%, P=0.0006) cell lines compared with the non-transfected cell lines and the negative control (Fig .4A-D). The data showed that antagomir-19a could increase myeloma cell susceptibility to drug-induced apoptosis.

### *SOCS3* and *STAT3* mRNA expression levels after transfection of myeloma cell lines with the LentimiRa-off-has-miR-19a-3p vect

**Fig.5:**
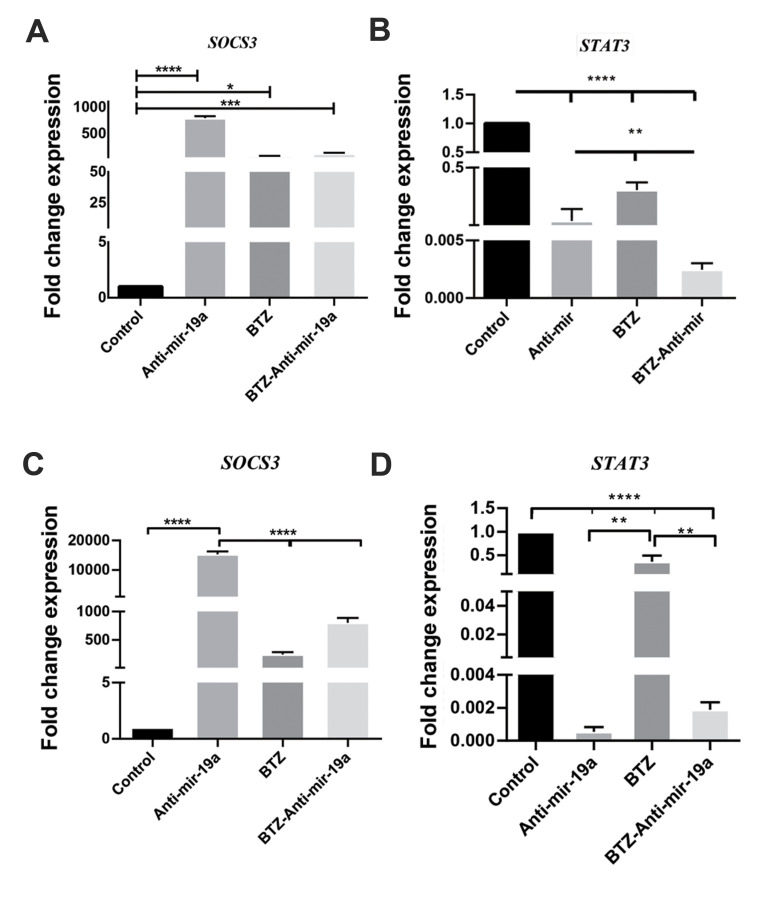
*SOCS3* and *STAT3* gene expression analyses at the mRNA level in RPMI 8226 and U266 cell lines by quantitative real-time polymerase chain reaction (qRT-PCR). The RPMI 8226 and U266 cells were cultured and treated with bortezomib (BTZ) (150 nM for RPMI 8226 and 5 µM for U266), transfected with the LentimiRa-off-has-mir-19a-3p vector and treated with BTZ (150 nM and 5 µM, respectively) after transfection. **A, B. **Expressions of *SOCS3* and *STAT3* were examined in RPMI 8226 cells after 48 hours of BTZ treatment and transfection with the LentimiRa-off-has-mir-19a-3p vector and after 48 hours of BTZ treatment in the transfected cells. **C, D.** *SOCS3* and *STAT3* expressions were examined in U266 cells after 48 hours of BTZ treatment and transfection with the LentimiRa-off-has-mir-19a-3p vector and after 48 hours of BTZ treatment in the transfected cells. Untreated RPMI 8226 and U266 cells were used as the controls to evaluate the relative gene expressions. The data are presented as mean ± SD of three independent experiments. *β-Actin* was the control in the qRT-PCR assessment. *; P<0.05, **; P<0.01, ***; P<0.001, and ****; P<0.0001 vs. the control.

*SOCS3* is a target of miR-19a. Therefore, we evaluated mRNA levels of *SOCS3* and its target, *STAT3*, in the myeloma cell lines after transfection with the Lentini-off-has-mir-19a-3p vector. There was an increase in mRNA expression of *SOCS3* and a decrease in *STAT3* mRNA expression compared with the non-transfected cell lines (negative control) (Fig .5A-D). 

### Anti-miR-19a induced downregulation of *PTEN, BCL-2*, and *CDKN1A* in the bortezomib-treated myeloma cell lines

**Fig.6:**
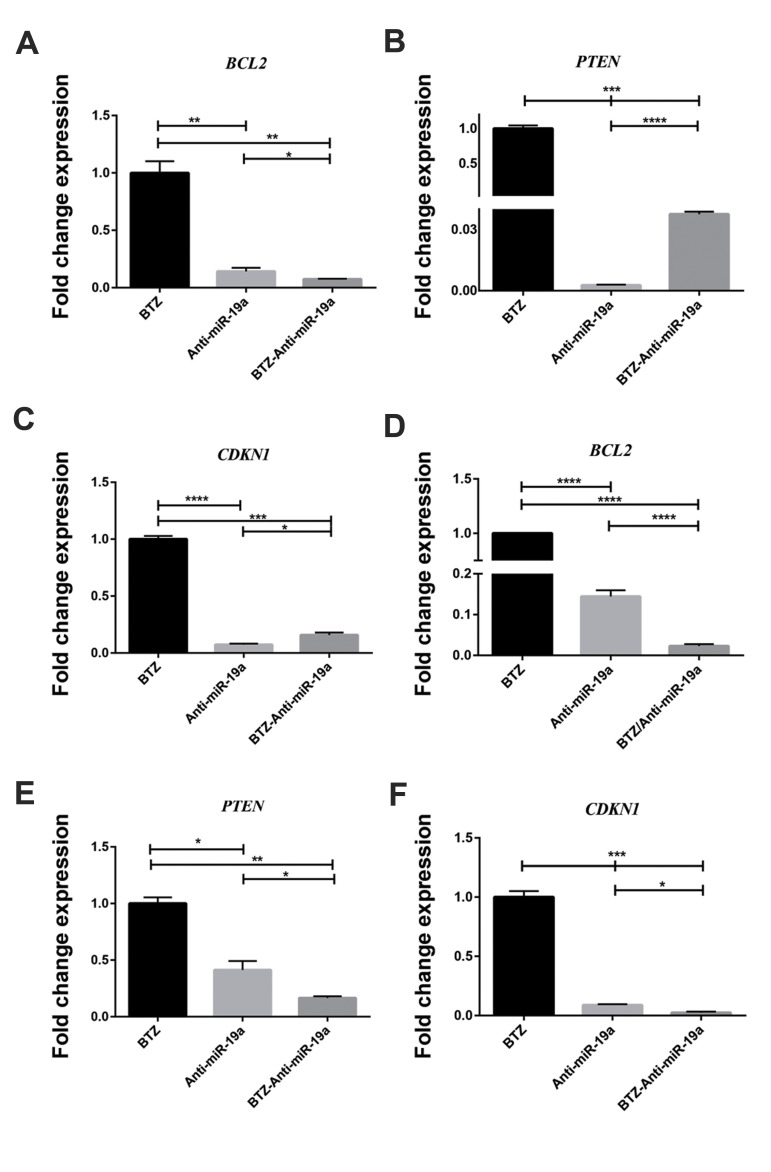
B-cell lymphoma 2 (*BCL-2*), *PTEN* and *CDKN1* expressions at the mRNA level in the U266 and RPMI 8226 cell lines according to quantitative real-time polymerase chian reaction (qRT-PCR) analysis. The U266 and RPMI 8226 cells were cultured and treated with bortezomib (BTZ), 5 µM and 150 nM, respectively, transfected with the LentimiRa-off-has-mir-19a-3p vector and treated with BTZ (5 µM and 150 nM, respectively) after transfection. **A-C**. Expressions of *BCL-2, PTEN* and *CDKN1* were examined in U266 cells after 48 hours of BTZ treatment and transfection with LentimiRa-off-has-mir-19a-3p vector, and after 48 hours of BTZ treatment in the transfected cells. **D-F.** Expressions of *BCL-2, PTEN *and *CDKN1* were examined in RPMI 8226 cells after 48 hours of BTZ treatment and transfection with LentimiRa-off-has-mir-19a-3p vector, and after 48 hours of BTZ treatment in the transfected cells. The U266 and RPMI 8226 cells treated with BTZ were used as the controls to evaluate the relative gene expressions. The data are presented as mean ± SD of three independent experiments. *β-Actin* served as the control for the qRT-PCR assessment. *; P<0.05, **; P<0.01, ***; P<0.001, and ****; P<0.0001 vs. the control.

Transfection of the LentiviRal-off-has-miR-19a-3p vector caused a decrease in *PTEN, BCL-2* and *CDKN1A* mRNA expressions in the BTZ-treated cell lines, which was consistent with downregulation of miR-19a after transfection compared to the BTZ-treated non-transfected cell lines (Fig .6).

## Discussion

Based on findings of previous studies (15), the first outcome of deregulated miRNA expression in MM was reported by Löffler et al. (16) when they reported that miR-21 ectopic expression made MM cells independent from IL-6 growth stimulus. Pichiorri et al. (17) identified a miRNA signature associated with transformation of normal PCs to clinical MM via monoclonal gammopathy of undetermined significance (MGUS) and demonstrated that miR-32, miR-21, miR-17-92, the miR-106b-25 cluster, and miR-181a/b upregulated in both MM cell lines and primary tumours versus normal PCs. Among these, the miR-17-92 cluster was only highly expressed in patients with MM. OncomiR-19a, one of the members of the miR-17-92 cluster, plays a role in promotion of cell proliferation, migration, and induction of apoptosis, and it is suggested to have a critical role in myeloma pathogenesis (10). Patients with low levels of miR-19a in their sera have a better response and shortened progression-free with downregulation of it obtained from others studies (18). Here, we evaluated the response of two MM cell lines to Bof the *JAK/STAT* pathwayTZ in the presence of antagomiR-19a in an attempt to avoid resistance to MM through targets of miR-19a. The results of recent studies have shown that *SOCS3* is a target of miR-19a and a negative regulator of *SOCS3* (19, 20), and showed that the molecule beneath it, *STAT3* is a significant promoter of cancers such as MM when activated (5, 21, 22). *STAT3*, one of the components , acts as an oncogene in human cancers (22, 23). Some miRNAs have close relativity with drug resistance; for example, it has been shown that miR-181a expression is in consistence with MM tumour load and could be a biomarker for treatment monitoring, as much as miR-20a, which is a potential diagnostic biomarker (4, 6). BTZ is a proteasome inhibitor and an effective treatment for MM in some patients; however, drug resistance is a major problem for 60% of these patients (8, 24). Our data primarily showed that antagomir-19a downregulated miR-19a expression. BTZ inhibited miR-19a in a concentration-dependent manner and, in the presence of antagomir-19a and BTZ, miR-19a efficiently downregulated in parallel with an increase in the proportion of apoptotic cells following treatment with BTZ. On the other hand, our viability studies showed that the ratio of proliferation of myeloma cell lines decreased after transfection with the antagomiR-19a vector. The data supported the results of other studies where miR-181a and miR-20a were inhibited by BTZ (6). 

We observed downregulation of *STAT3* and upregulation of *SOCS3* at the mRNA level, which confirmed that miR-19a is a negative regulator of *SOCS3*. This finding supported data from previous studies (19, 20). The oncogenic function of *STAT3* has been previously reported (21, 22); therefore, we could conclude that inhibition of miR-19a caused suppression of *STAT3*. Our results showed that the expression level of *STAT3* in BTZ-treated cells highly decreased when used in parallel with anatago-miR-19a. Thus, inhibition of miR-19a could be used to improve BTZ responsiveness in myeloma cells. The results of another study showed that *PTEN* plays critical roles in regulating cell proliferation, differentiation and apoptosis, and a molecular study identified *PTEN* as a downstream target of miR-19a, which was inversely correlated with miR-19a expression in ovarian cancer tissues (10). We also assessed *BCL-2*, which is an attractive target for therapy. In this context, there is a drug that targets *BCL-2* in *BCL-2*-dependent haematologic malignancies, such as chronic lymphoid leukaemia and mantle cell lymphoma (11, 25). In a recent study, miR-19a promoted drug resistance of myeloma cells to chemotherapeutic agents by upregulation of *BCL-2* (11). A previous study indicated that *CDKN1* is an oncotarget in Burkitt lymphoma and MM. Currently, *CDKN1* is more broadly considered to be a regulator of fundamental cell-fate decisions such as proliferation, differentiation, and senescence (13).

In accordance with these studies, our data showed that inhibition of miR-19a by the antagomiR-19a and BTZ treatment of cells increased the expression levels of *PTEN, BCL-2*, and *CDKN1*. However, their expressions decreased in myeloma cell lines treated with BTZ after induction of antagomiR-19a. The results supplement the findings that miRNAs are differentially expressed in BTZ-resistant myeloma cells. miR-19a could be a possible prognostic biomarker for responsiveness to BTZ in MM patients. In order to overcome resistance and improve the level of responsiveness to BTZ, miR-19a targets such as *SOCS3* and *STAT3* could be tracked. We faced some restrictions in our studies that must be adverted. They include the use of other MM cell lines and myeloma cells obtained from MM patients, and evaluation of targets at the protein level expression and downstream molecules, which could have helped us to generalize our results.

## Conclusion

Overall, our data indicated that induction of antagomir-19a in myeloma cell lines resulted in downregulation of mir-19a and enhanced responsiveness to BTZ treatment. On the other hand, the ratio of apoptosis in the BTZ-treated cell lines was drastically more effective in the presence of mir-19a inhibition. Thus, mir-19a could be a potential prognostic biomarker in MM treatment. Downregulation of the *PTEN* and *CDKN1* oncogenes due to inhibition of oncomiR-19a following BTZ treatment would most likely lead to a better treatment response.
